# Pancancer analysis of the prognostic and immunological role of *FANCD2*: a potential target for carcinogenesis and survival

**DOI:** 10.1186/s12920-024-01836-4

**Published:** 2024-03-05

**Authors:** Zedan Zhao, Ruyu Wang, Ruixue Wang, Jialing Song, Fengjun Ma, Huafeng Pan, Cuiyun Gao, Deqiang Wang, Xuemei Chen, Xiangzhen Fan

**Affiliations:** 1 Department of Rehabilitation Medicine, Binzhou Medical University Hospital, Binzhou, Shandong 256603 China; 2 School of Rehabilitation Medicine, Binzhou Medical University, Yantai, Shandong 264003 China; 3 Department of Obstetrics, Binzhou Medical University Hospital, Binzhou, Shandong 256603 China; 4 School of clinical medicine, Jiangxi University of Chinese Medicine, Nanchang, Jiangxi 330004 China; 5 Shandong University of Traditional Chinese Medicine, Jinan, Shandong China; 6 Science and Technology Innovation Center, Guangzhou University of Chinese Medicine, Guangzhou, Guangdong China

**Keywords:** *FANCD2*, Pan-cancer analysis, Prognosis, Immune infiltration, Genetic alteration, Enrichment analysis

## Abstract

Recent evidence has shed light on the significant role of *FANCD2* in cancer initiation, development, and progression. However, a comprehensive pan-cancer analysis of *FANCD2* has been lacking. In this study, we have conducted a thorough investigation into the expression profiles and prognostic significance of *FANCD2*, as well as its correlation with clinicopathological parameters and immune cell infiltration, using advanced bioinformatic techniques. The results demonstrate that *FANCD2* is significantly upregulated in various common cancers and is associated with prognosis. Notably, higher expression levels of *FANCD2* are linked to poor overall survival, as indicated by Cox regression and Kaplan-Meier analyses. Additionally, we have observed a decrease in the methylation of *FANCD2* DNA in some cancers, and this decrease is inversely correlated with *FANCD2* expression. Genetic alterations in *FANCD2* predominantly manifest as mutations, which are associated with overall survival, disease-specific survival, disease-free survival, and progression-free survival in certain tumor types. Moreover, *FANCD2* exhibits a strong correlation with infiltrating cell levels, immune checkpoint genes, tumor mutation burden (TMB), and microsatellite instability (MSI). Enrichment analysis further highlights the potential impact of *FANCD2* on Fanconi anemia (FA) pathway and cell cycle regulation. Through this comprehensive pan-cancer analysis, we have gained a deeper understanding of the functions of *FANCD2* in oncogenesis and metastasis across different types of cancer.

**Supplementary Information:**

The online version contains supplementary material available at 10.1186/s12920-024-01836-4.

## Introduction

In recent years, the incidence and mortality rates of malignant tumors have shown a significant increase [1, 2]. A study has estimated that the global economic burden of cancer treatment could amount to approximately $25.2 trillion between the years 2020 and 2050 [3]. It is worth noting that the impact of cancer extends beyond its economic implications, encompassing profound societal consequences [4, 5]. Despite remarkable advancements in surgical procedures, radiotherapy, adjuvant chemotherapy, immunotherapy, and targeted therapies that have revolutionized the field of cancer management, the overall prognosis for cancer patients remains disheartening, with the five-year survival rate remaining far from satisfactory [6–8]. Consequently, there exists an imperative to actively explore innovative approaches in the realms of cancer diagnosis and treatment. As a result, the relentless pursuit of novel avenues and tumor biomarkers for the purposes of cancer detection and intervention becomes paramount.

Fanconi anemia (FA) is an inherited disorder of DNA instability resulting from mutually affected mutations within one of the 22 *FANC* genes along the FA pathway, an intricate network intimately involved in the mending of DNA damage and the orchestration of stress response [9, 10]. FA patients exhibit a precocious manifestation of senescence, profound aplasia of the bone marrow, and an exceptionally elevated susceptibility to diverse malignancies [11, 12]. The monoubiquitination of FANCD2 plays a crucial role in its chromatin localization and the formation of DNA damage repair foci, serving as the pivotal event in the DNA repair mechanism governed by the FA pathway [13]. In addition to its proficiency in repairing DNA damage, *FANCD2* also assumes pivotal roles in the progression of cancer. An elevated magnitude of *FANCD2* expression, concomitant with the unfavorable prognostic outlook of patients, has been detected in cases of endometrial cancer [14], hepatocellular carcinoma [15] and ovarian carcinomas [16]. However, the comprehensive understanding of *FANCD2’*s role in various cancer types remains limited.

Recently, pan-cancer analysis has revolutionized cancer research by allowing the analysis of gene expression, regulation, prognosis, and mutations across diverse tumor types in a unified manner [17]. This approach provides valuable insights into the molecular pathobiology of cancer and facilitates the development of novel directions and strategies for clinical cancer treatment. Given the significance of pan-cancer analysis, the objective of this study was to comprehensively investigate the expression of *FANCD2* across multiple cancer types. This investigation involved searching various publicly available databases to assess its diagnostic, prognostic, and immune infiltration characteristics in pan-cancer cohorts. These findings serve as a foundation for identifying and evaluating novel immunotherapeutic targets associated with *FANCD2* and elucidating the specific mechanisms underlying *FANCD2’*s involvement in cancer progression.

## Materials and methods

### Gene and protein expression analysis

The expression differences of *FANCD2* between normal and tumor tissues were analyzed using TIMER2.0 (http://timer.cistrome.org/), which is a tool based on the TCGA database (https://portal.gdc.com). Gene expression levels were measured as log2 TPM values, and RNA-sequencing expression profiles (level 3) along with clinical information for *FANCD2* were obtained from the TCGA dataset. The analysis was conducted using R version 4.0.3, and the Wilcox test was used to analyze the two-group data. A significance level of *P* < 0.05 was used to determine statistical significance. The study included 33 cancer types, which are as follows: adrenocortical carcinoma (ACC), bladder urothelial carcinoma (BLCA), breast invasive carcinoma (BRCA), cervical squamous cell carcinoma and endocervical adenocarcinoma(CESC), cholangiocarcinoma (CHOL), colon adenocarcinoma (COAD), lymphoid neoplasm diffuse large B-cell lymphoma (DLBC), esophageal carcinoma (ESCA), glioblastoma multiforme (GBM), head and neck squamous cell carcinoma (HNSC), kidney chromophobe (KICH), kidney renal clear cell carcinoma (KIRC), kidney renal papillary cell carcinoma (KIRP), acute myeloid leukemia (LAML), brain lower grade glioma (LGG), liver hepatocellular carcinoma (LIHC), lung adenocarcinoma (LUAD), lung squamous cell carcinoma (LUSC), mesothelioma (MESO), Ovarian serous cystadenocarcinoma (OV), pancreatic adenocarcinoma (PAAD), pheochromocytoma and paraganglioma (PCPG), prostate adenocarcinoma (PRAD), rectum adenocarcinoma(READ), sarcoma (SARC), skin cutaneous melanoma (SKCM), stomach adenocarcinoma (STAD), testicular germ cell tumors (TGCT), thyroid carcinoma (THCA), thymoma (THYM), uterine corpus endometrial carcinoma (UCEC), uterine carcinosarcoma (UCS), uveal melanoma (UVM). The disparities in overall protein expression levels of FANCD2 between normal and tumor tissues were obtained from the CPTAC database via the UALCAN tool (http://ualcan.path.uab.edu/analysis-prot.html). We utilized UALCAN to investigate the association between FANCD2 expression and tumor staging. The immunohistochemical (IHC) images of human normal tissues and tumor tissues were obtained from the Human Protein Atlas (HPA, https://www.proteinatlas.org/).

### Survival prognosis analysis

We obtained RNA-sequencing expression profiles (level 3) and relevant clinical information for 33 different types of cancer from the TCGA dataset. For analysis, we utilized univariate Cox regression analysis and generated a forest plot using the “forestplot” R package to display the *P* value, hazard ratio (HR), and 95% confidence interval (CI) for each variable. All the statistical analyses and R packages were implemented using R version 4.0.3. Unless mentioned otherwise, two-group data were compared using the Wilcox test. Significance was defined as a *P* value less than 0.05.

We utilized the Kaplan-Meier plotter (http://www.kmplot.com) to assess the relationship between *FANCD2* expression and the prognosis of cancer patients. This online tool allowed us to analyze the survival data and investigate the impact of *FANCD2* expression on patient outcomes.

### Immune infiltration and immune checkpoint analysis

We downloaded RNA-sequencing expression profiles (level 3) and corresponding clinical information for 33 types of cancer from the TCGA dataset. To ensure reliable results in immune score evaluation, we utilized the immuneeconv software package. This comprehensive R package integrates the latest algorithms, including TIMER, xCell, MCP-counter, CIBERSORT, EPIC, and quantized, to assess immune scores. We focused on transcripts associated with immune checkpoints, namely SIGLEC15, IDO1, CD274, HAVCR2, PDCD1, CTLA4, LAG3, and PDCD1LG2. We extracted the expression values of these eight genes and investigated their association with immune checkpoint activity. All data analysis methods and R packages were implemented using R version 4.0.3. Unless specified, comparisons between two groups of data were performed using the Wilcox test. A *P* value of less than 0.05 was considered statistically significant.

### TMB and MSI analysis

We obtained RNA-sequencing expression profiles (level 3) and corresponding clinical information for 33 types of cancer from the TCGA dataset. The calculation of tumor mutation burden (TMB) was based on the research article “The Immune Landscape of Cancer” published by Vesteinn Thorsson et al. in 2018 [18] while the microsatellite instability (MSI) data was derived from the article “Landscape of Microsatellite Instability Across 39 Cancer Types” published by Russell Bonneville et al. in 2017 [19]. All statistical analyses and R package implementations were conducted using R version 4.0.3. Unless explicitly specified, comparisons between two groups of data were performed using the Wilcox test. A *P* value of less than 0.05 was considered statistically significant.

### Gene enrichment analysis

We utilized the STRING tool (https://cn.string-db.org/) to download the top 50 proteins related to FANCD2, and a protein-protein interaction (PPI) network was constructed using Cytoscape. The top 100 genes correlated with *FANCD2* were obtained, and for the top five genes, Pearson’s correlation test was performed using GEPIA2. To visualize the correlations, a heatmap of the top five genes was generated using TIMER2.0. Additionally, we identified eight genes that interacted between the two databases, and a Venn diagram was created using the Draw Venn Diagram tool (http://bioinformatics.psb.ugent.be/webtools/Venn/). To gain further insights, we input the combined targets from the two databases into R 4.3.1 software and conducted gene ontology (GO) and Kyoto Encyclopedia of Genes and Genomes (KEGG) enrichment analysis using the “clusterProfiler” package. The criteria for significant enrichment were set as a *P*-value < 0.05 and a Q-value < 1.

### Genetic alteration analysis

We applied the UALCAN tool to investigate the DNA methylation levels of *FANCD2* across different cancer tissues, in comparison to their corresponding normal tissues. We utilized the cBioPortal tool (http://www.cbioportal.org/) to analyze the genetic variations in *FANCD2*. The alteration frequency, mutation site, and survival data were obtained by selecting the options “cancer types summary,” “mutations,” and “comparison/survival” in the query module.

### Correlation analysis between genes and pathways

We obtained RNA-sequencing expression profiles (level 3) and relevant clinical information for 33 cancer types from the TCGA dataset. To analyze the data, we utilized the GSVA package in R software, with the parameter set as method=‘ssgsea’. Spearman correlation was employed to assess the correlation between *FANCD2* and DNA damage repair-pathway scores. All analyses were performed using R version 4.0.3. A *p*-value less than 0.05 was considered statistically significant.

## Results

### Pan-cancer analysis of *FANCD2* expression

**Fig. 1:**
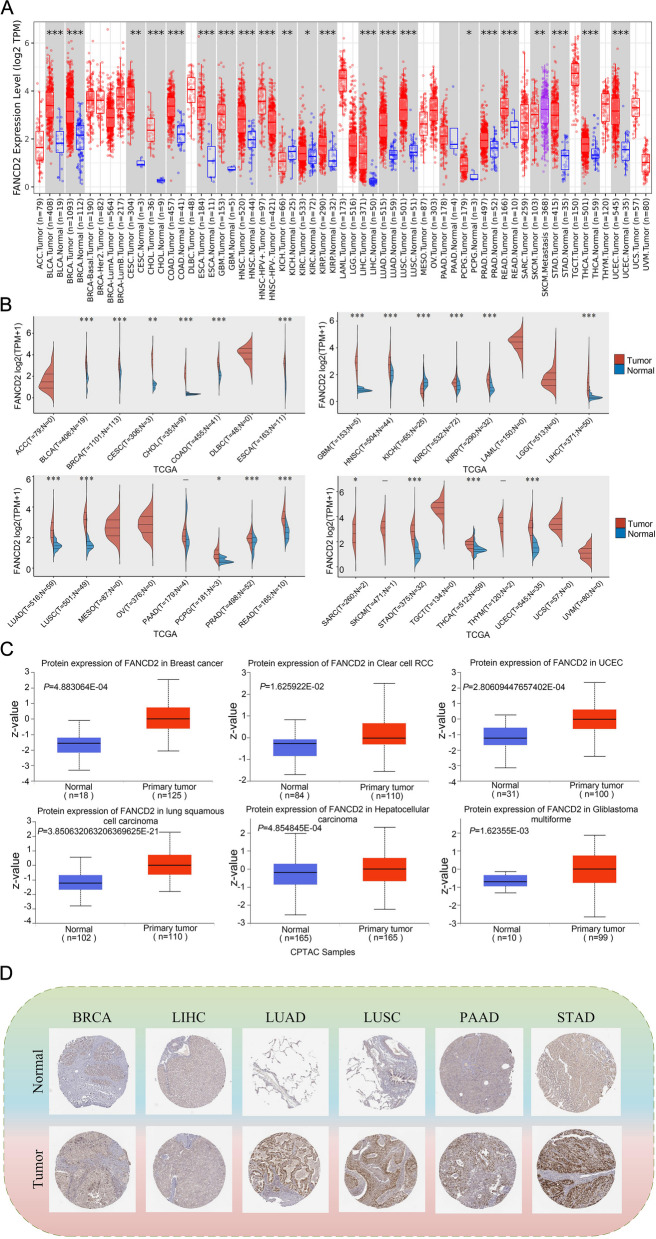
Expression of the * FANCD2* gene in pan-cancer. **A** The TIMER database was used to analyze the expression of the *FANCD2* gene in different cancers or specific cancer sub-types. **B** The expression distribution of the *FANCD2* gene in tumor and normal tissues from TCGA. **C** The CPTAC dataset was used to analyze the protein expression level of FANCD2 in normal and primary tissues of breast cancer, RCC, UCEC, LUSC, LIHC, and GBM. **D** The IHC images of FANCD2 in normal and tumor tissues extracted from the HPA. *P < 0.05, **P < 0.01, and ***P < 0.001

The TIMER database was utilized to analyze the expression of *FANCD2* in both cancerous and normal tissues. Our findings revealed that *FANCD2* expression was significantly higher in tumor tissues compared to normal tissues in several datasets including BLCA, BRCA, CESC, CHOL, COAD, ESCA, GBM, HNSC, KICH, KIRC, KIRP, LIHC, LUAD, LUSC, PCPG, PRAD, READ, STAD, THCA, and UCEC (Fig. 1A). In addition, the TCGA data also confirmed a noticeable upregulation of *FANCD2* mRNA expression in BLCA, BRCA, CESC, CHOL, COAD, ESCA, GBM, HNSC, KICH, KIRC, KIRP, LIHC, LUAD, LUSC, PCPG, PRAD, READ, SARC, STAD, THCA, and UCEC compared to normal tissues (Fig. 1B), supporting the findings from the TIMER database. Further analysis using the CPTAC database showed a significant increase in FANCD2 protein expression in breast cancer, RCC, UCEC, LUSC, LIHC, and GBM (Fig. 1C). To validate the protein expression of FANCD2, we obtained the IHC results of FANCD2 in pan-cancer from the HPA database. As shown in Fig. 1D, The staining intensity of FANCD2 was higher in the nuclei of BRCA, LIHC, LUAD, LUSC, PAAD, and STAD cells, which was consistent with the results of mRNA expression level analysis of *FANCD2*. Overall, *FANCD2* was highly expressed in most cancers.

### Pan-cancer analysis of the relationship between *FANCD2* expression and clinicopathology

**Fig. 2:**
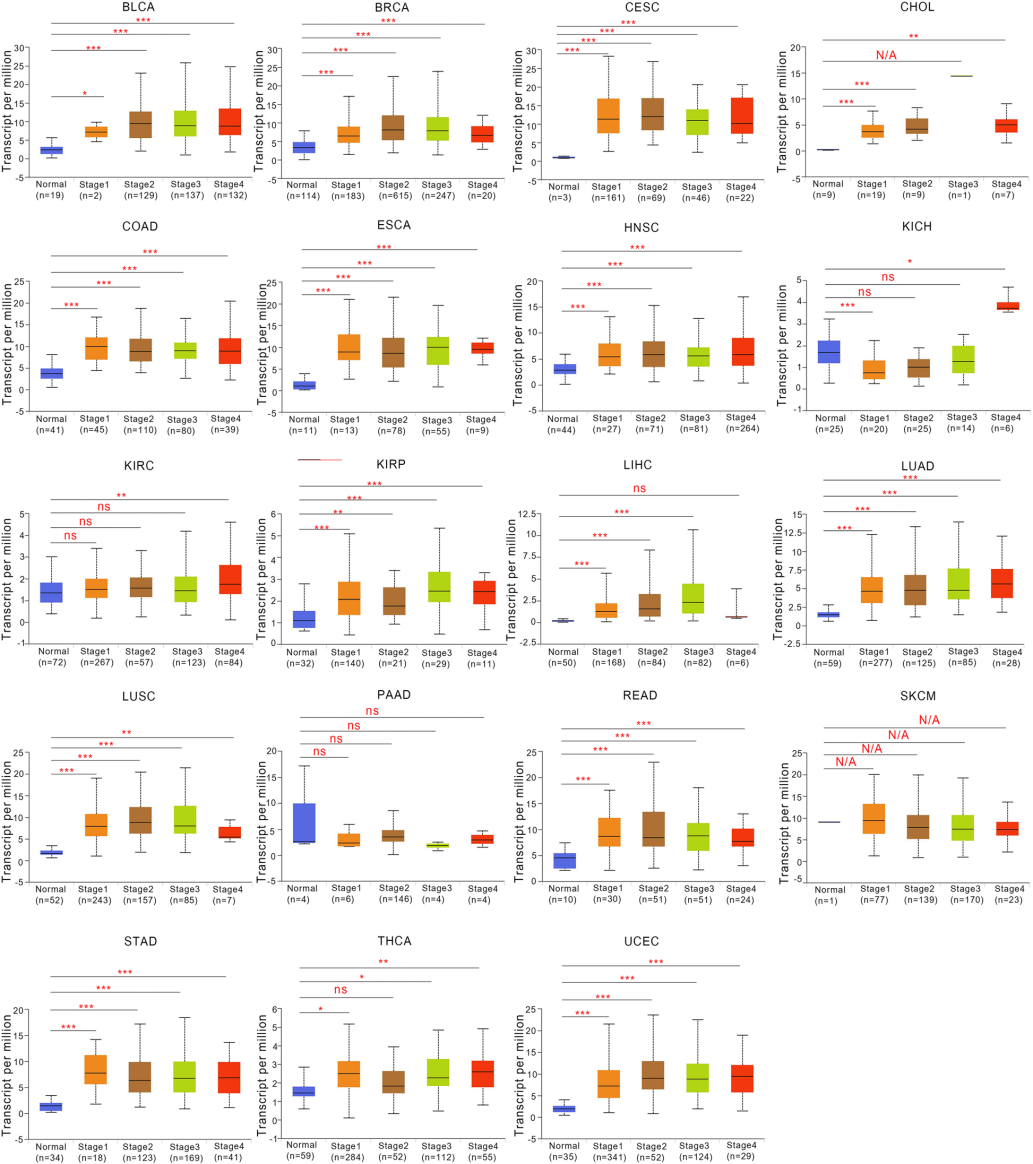
Correlations between *FANCD2* gene expression and the main pathological stages of BLCA, BRCA, CESC, CHOL, COAD, ESCA, HNSC, KICH, KIRC, KIRP, LIHC, LUAD, LUSC, PAAD, READ, SKCM, STAD, THCA, and UCEC based on the TCGA dataset. *P < 0.05, **P < 0.01, ***P < 0.001, ns No significance, and N/A not applicable

We investigated the expression patterns of *FANCD2* in tumor tissues across various stages (I to IV) of multiple cancers using the TCGA database. Our aim was to explore the potential correlation between *FANCD2* expression and clinicopathological features. The results presented in Fig. 2 indicate that the expression of *FANCD2* significantly increases during disease progression, starting from normal tissue and advancing to early malignancy and ultimately to the terminal stage, in several cancers including BLCA, BRCA, CESC, CHOL, COAD, ESCA, HNSC, KICH, KIRC, KIRP, LIHC, LUAD, LUSC, READ, STAD, THCA and UCEC. However, in some cancers such as PAAD, *FANCD2* expression is consistent throughout disease progression.

### Pan-cancer analysis of the prognostic value of *FANCD2*

**Fig. 3:**
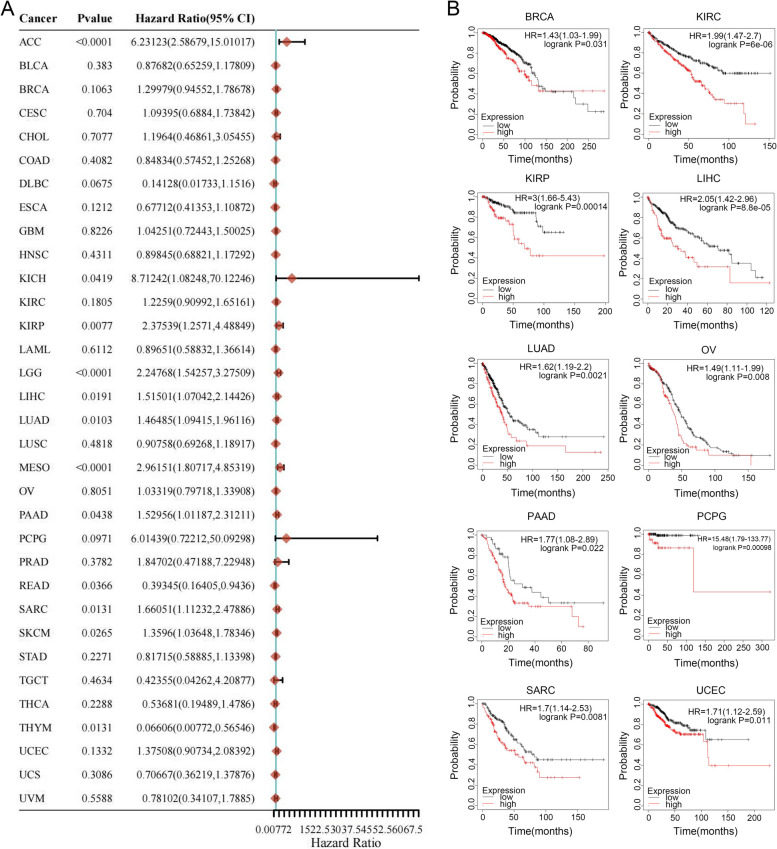
Association between *FANCD2* expression and the OS of cancer patients. **A** A forest plot displaying hazard ratios of *FANCD2* in 33 types of tumors. **B** Kaplan-Meier survival curves illustrating the OS of patients categorized by the varying expressions of *FANCD2* in BRCA, KIRC, KIRP, LIHC, LUAD, OV, PAAD, PCPG, SARC, and UCEC. *P < 0.05, **P < 0.01, and ***P < 0.001

We conducted Cox proportional hazards model and Kaplan-Meier analysis to investigate the prognostic value of *FANCD2* across a range of cancer types. The results of our Cox regression analysis revealed a significant correlation between *FANCD2* expression and overall survival (OS) in 12 specific cancer types, namely ACC, KICH, KIRP, LGG, LIHC, LUAD, MESO, PAAD, READ, SARC, SKCM, and THYM (Fig. 3A). Moreover, the Kaplan-Meier survival curves exhibited a strong association between increased *FANCD2* expression and poorer OS in BRCA, KIRC, KIRP, LIHC, LUAD, OV, PAAD, PCPG, SARC, and UCEC, whereas no statistical differences existed in other tumors (Fig. 3B). These data indicate that the prognostic impact of *FANCD2* expression is specifically associated with the type of cancer, highlighting its potential as a differential prognostic marker in different tumor contexts.

### Pan-cancer analysis of the immunological role of *FANCD2*

**Fig. 4:**
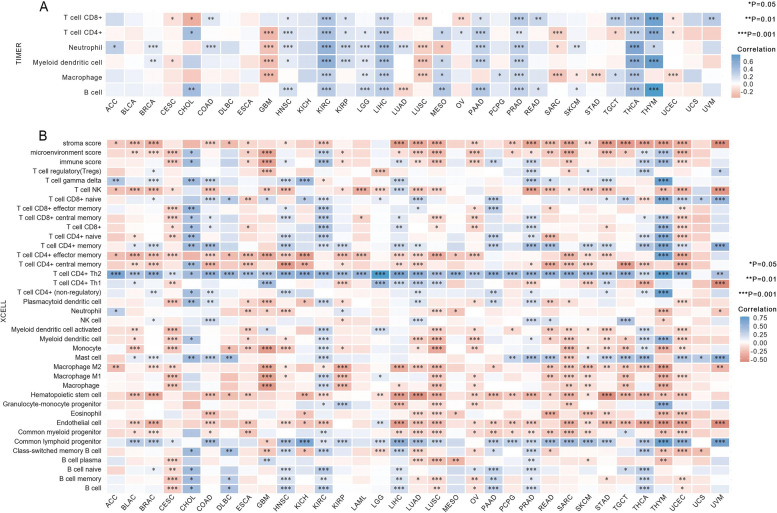
Correlation between *FANCD2* expression and immune infiltration. **A** The correlation between *FANCD2* expression and the infiltration levels of various immune cells in the TIMER database. **B** The correlation between *FANCD2* expression and the infiltration levels of various immune cells based on xCell. *P < 0.05, **P < 0.01, and ***P < 0.001

It is well recognized that immune cells play a crucial role in shaping the immune microenvironment and can have a significant impact on the prognosis of cancer patients [20]. In our study, we conducted a comprehensive pan-cancer analysis using the TIMER database to investigate the association between *FANCD2* expression and the infiltration of six immune-related cell types. As depicted in Fig. 4A, we observed a significant correlation between *FANCD2* expression and the abundance of infiltrating immune cells in various cancer types. Specifically, *FANCD2* expression showed a significant correlation with the abundance of CD8 + T cells in 17 types of cancer, CD4 + T cells in 16 types of cancer, neutrophils in 18 types of cancer, myeloid dendritic cells in 14 types of cancer, macrophages in 14 types of cancer, and B cells in 13 types of cancer. Moreover, we utilized the xCell online tool to further investigate the relationship between *FANCD2* expression and the infiltration of different immune cell subtypes. Among the 38 scrutinized subsets of immune cells, we observed a direct correlation between *FANCD2* expression and the degree of Th2 CD4 + T-cell infiltration across 32 diverse cancer types. In most cases, *FANCD2* exhibited either a positive or negative association with the infiltration of the remaining 37 immune cell subsets (Fig. 4B).

### Pan-cancer analysis of the correlation between the *FANCD2* expression and immune checkpoint genes

**Fig. 5:**
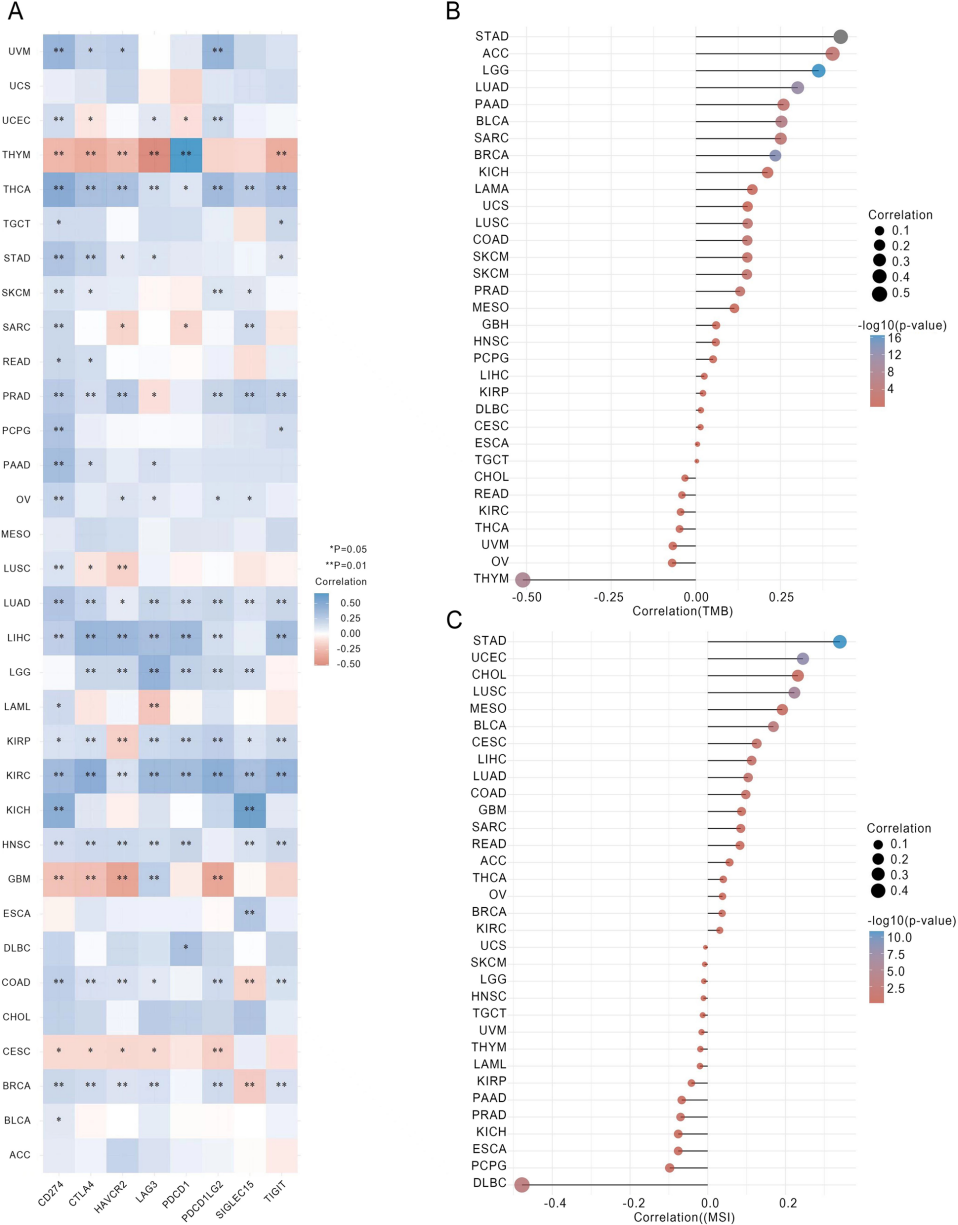
Correlation of *FANCD2* expression with pan-cancer immune checkpoints, TMB and MSI. **A** Correlation between *FANCD2* expression and immune checkpoint genes in pan-cancer. **B** Correlation between *FANCD2* expression and TMB in pan-cancer. **C** Correlation between *FANCD2* expression and MSI in pan-cancer. *P < 0.05, **P < 0.01, and ***P < 0.001

The immune checkpoints play a critical role in maintaining immune system balance and preventing the development of malignancies and autoimmune diseases [21]. In our study, we investigated the relationship between *FANCD2* and immune checkpoints in 33 different tumors. The results demonstrated a positive correlation between *FANCD2* and immune checkpoints in most tumor tissues, including CTLA4, HAVCR2, LAG3, PDCD1, PDCD1LG2, SIGLEC15, TIGIT, and particularly CD274 (Fig. 5A). These findings suggest that *FANCD2* could be a potential target for immune therapy.

The determination of tumor mutational burden (TMB) and microsatellite instability (MSI) status is crucial in predicting a patient’s prognosis and deciding suitable treatment options [22]. Therefore, we further investigated the correlations between *FANCD2* expression and TMB as well as MSI. Our results showed that *FANCD2* expression was significantly positively correlated with TMB in STAD, ACC, LGG, LUAD, PAAD, BLCA, SARC, BRCA, LUSC, COAD, SKCM, and BRCA, and negatively correlated with TMB in THYM (Fig. 5B). Additionally, *FANCD2* expression was positively correlated with MSI in STAD, UCEC, LUSC, BLCA, CESC, LIHC, LUAD, and COAD, and negatively correlated in DLBC (Fig. 5C).

### Pan-cancer analysis of genetic alteration and DNA methylation of *FANCD2*

**Fig. 6:**
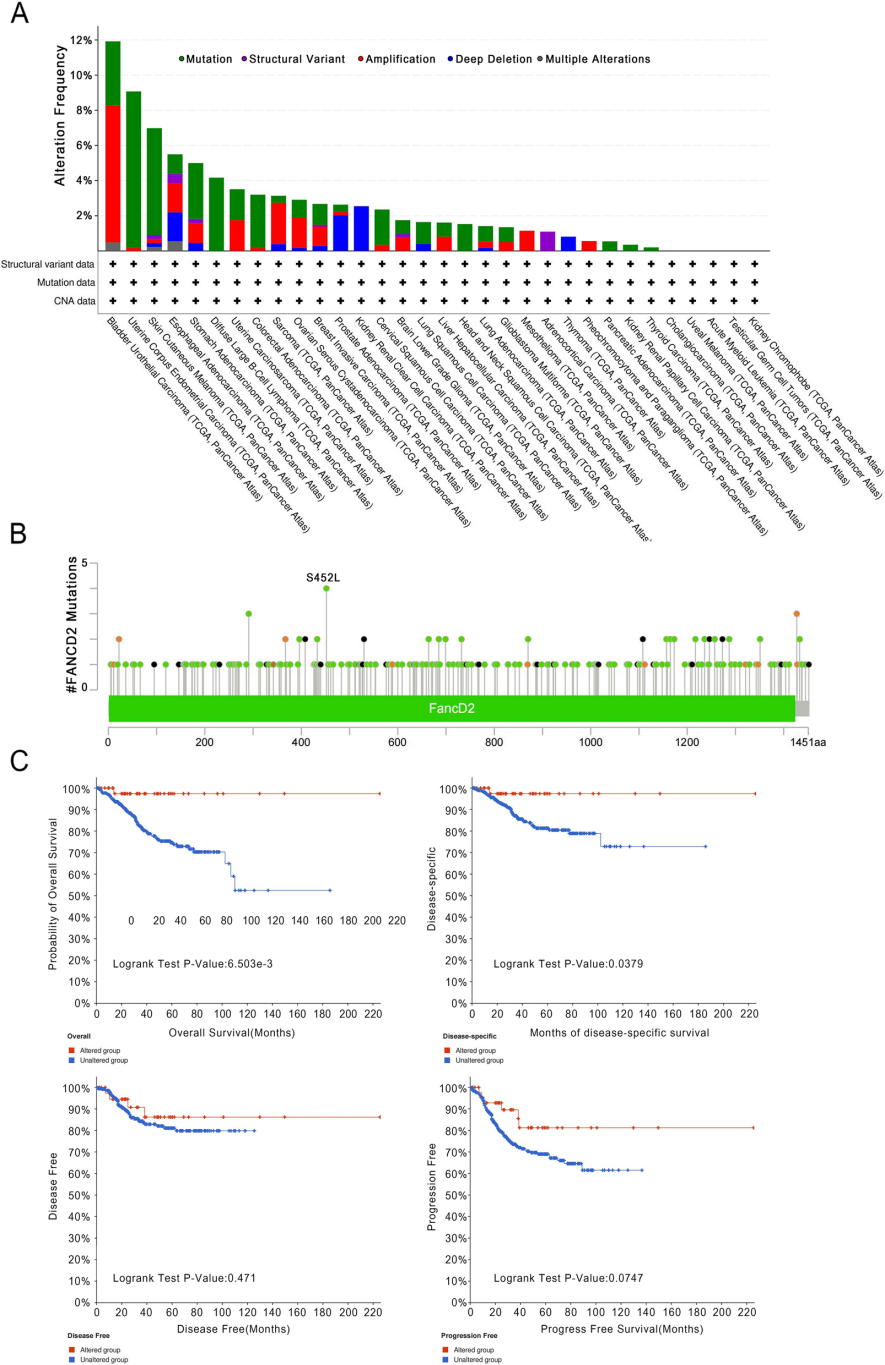
Genetic alteration features of *FANCD2* in pan-cancer. **A**, **B **The alteration frequency with different types of mutations in the cBioportal database. The mutation types, sites, and case numbers of *FANCD2* genetic alterations are shown below. **C** The effect of *FANCD2* mutation status on overall, disease-specific, disease-free, and progression-free survival of UCEC patients was investigated using the cBioPortal database. *P < 0.05, **P < 0.01, and ***P < 0.001

Cancer emerges as a consequence of genetic modifications, wherein the integrity of one or more genes is affected. These alterations manifest in various forms, encompassing mutations, structural variations, amplifications, profound deletions, and multiple modification [23]. The cBioPortal online tool was employed to examine the expression status of *FANCD2* across multiple cancer types. Our findings revealed that BLCA, UCEC, SKCM, ESCA, STAD, and DLBC exhibited the highest prevalence of *FANCD2* alterations. In addition, genetic modifications in *FANCD2* were primarily detected in the form of mutation, amplification, and deep deletion, as illustrated in Fig. 6A. Furthermore, we provided a comprehensive depiction of the specific type, location, and quantity of genetic alterations in *FANCD2* (Fig. 6B). Among the 241 mutation sites spanning amino acids 0 to 1451, we identified 179 missense mutations, 35 truncating mutations, 1 inframe mutation, 19 splices, and 7 fusion alterations. Notably, the mutation site S452L was the most frequently observed. In addition, we conducted an investigation into the association between genetic modifications in *FANCD2* and the prognosis of UCEC by utilizing the cBioPortal database. The findings revealed that individuals with modified levels of *FANCD2* displayed a more favorable prognosis in relation to both the overall and disease-specific survival, but not in disease-free and progress-free survival, in comparison to those lacking *FANCD2* alteration (Fig. 6C).

Defects in DNA methylation and its mediators can lead to genomic instability, and the presence of imperfections in the process of DNA methylation and its intermediaries can result in genomic instability, while one distinguishing feature of cancer cells lies in their intricate DNA methylation pattern [24, 25]. By utilizing the UALCAN database, we conducted an analysis comparing the levels of methylation in the *FANCD2* gene between both healthy and tumorous tissues. Our findings revealed a marked increase in *FANCD2* promoter methylation within ESCA, KIRC, and LUSC tumor tissues when compared to their respective normal counterparts. Conversely, a decrease in methylation was observed in UCEC (Supplementary Figure 1).

### Enrichment analysis of *FANCD2*-related genes

**Fig. 7:**
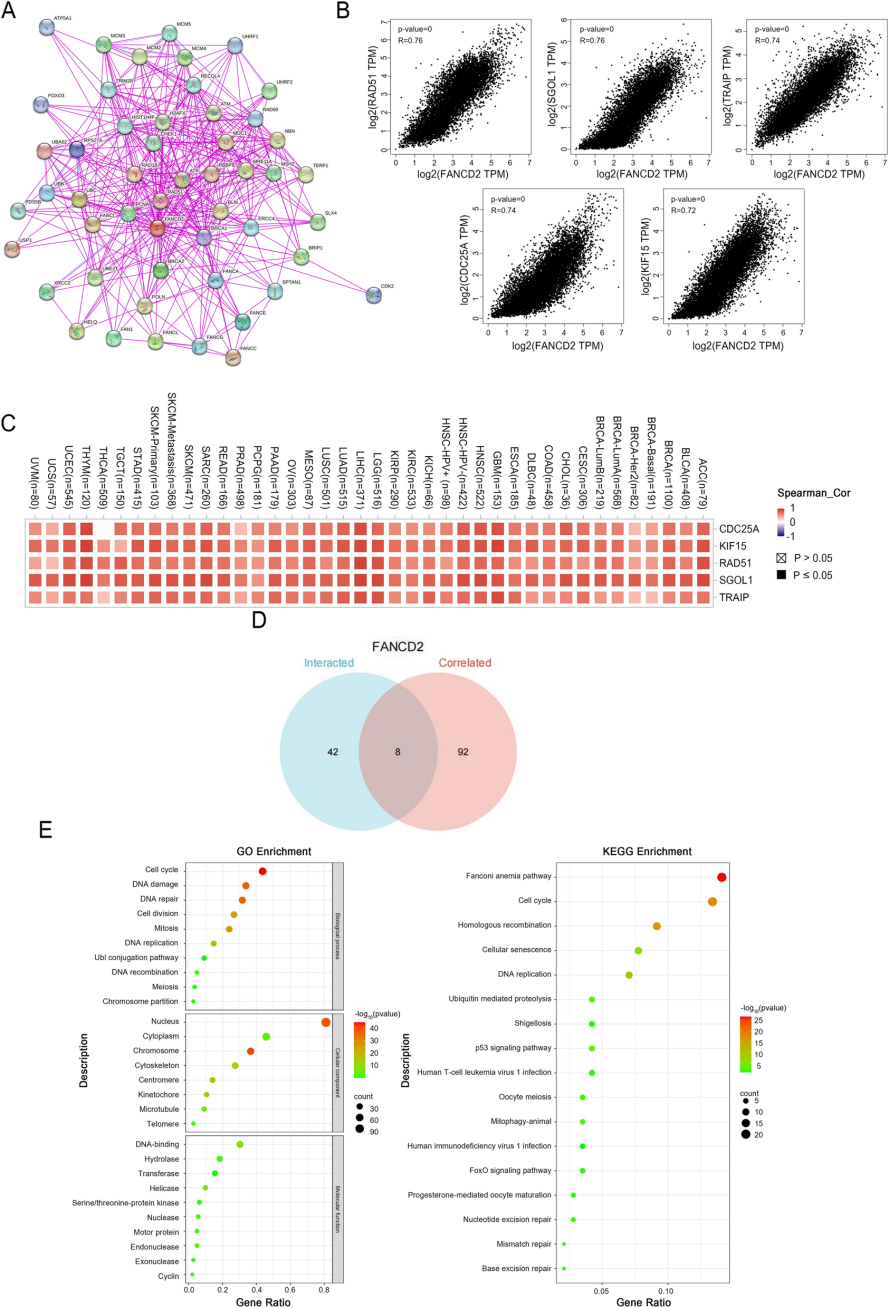
Enrichment analysis of *FANCD2*-related genes. **A** A PPI network of the top 50 FANCD2-related proteins was constructed using STRING data base. **B** Correlations between *FANCD2* expression and five specific genes (*RAD51*, *SGOL1*, *TRAIP*, *CDC25A*, and *KIF15*) in pan-cancer, along with the visualization of the corresponding heatmap data (**C**) using GEPIA2. **D** The top 100 *FANCD2*-correlated genes were obtained from TCGA projects and the interacted genes with the STRING data base were acquired. **E** GO and KEGG enrichment analysis of *FANCD2*-related genes

In order to further investigate the role of the *FANCD2* gene in tumorigenesis, our study aimed to identify FANCD2-binding proteins and *FANCD2* expression-correlated genes for pathway enrichment analyses. We successfully identified 50 FANCD2-binding proteins using the STRING tool and visualized their interaction network (Fig. 7A). The expression level of *FANCD2* showed a positive correlation with genes such as *RAD51* (*R* = 0.76), *SGOL1* (*R* = 0.76), *TRAIP* (*R* = 0.74), *CDC25A* (*R* = 0.74), and *KIF15* (*R* = 0.72) (Fig. 7B). The heat map analysis demonstrated this positive correlation across various cancer types (Fig. 7C). Additionally, using the GEPIA2 tool, we integrated tumor expression data from TCGA and identified the top 100 genes significantly correlated with *FANCD2* expression. An intersection analysis revealed eight common members, including *BRCA1*, *CHEK1*, *FANCI*, *MCM3*, *MSH2*, *RAD51*, *XRCC2*, and *UBE2T* (Fig. 7D). We further conducted KEGG and GO enrichment analyses on the combined datasets. The GO enrichment analysis indicated that these genes are mainly involved in DNA-related pathways and cellular biology (Fig. 7E). The KEGG enrichment analysis highlighted potential pathways, such as “Fanconi anemia pathway,” “Cell cycle,” “Homologous recombination,” “Cellular senescence,” “DNA replication,” “Human T-cell leukemia virus 1 infection,” “Shigellosis,” “Ubiquitin-mediated proteolysis,” and “*P53* signaling pathway,” which may play a role in the tumorigenic function of *FANCD2* (Fig. 7E). These findings provide insights into the molecular mechanisms of *FANCD2* in tumor development.

### Correlation analysis between *FANCD2* expression and DDR pathway and G2/M checkpoint pathway

**Fig. 8:**
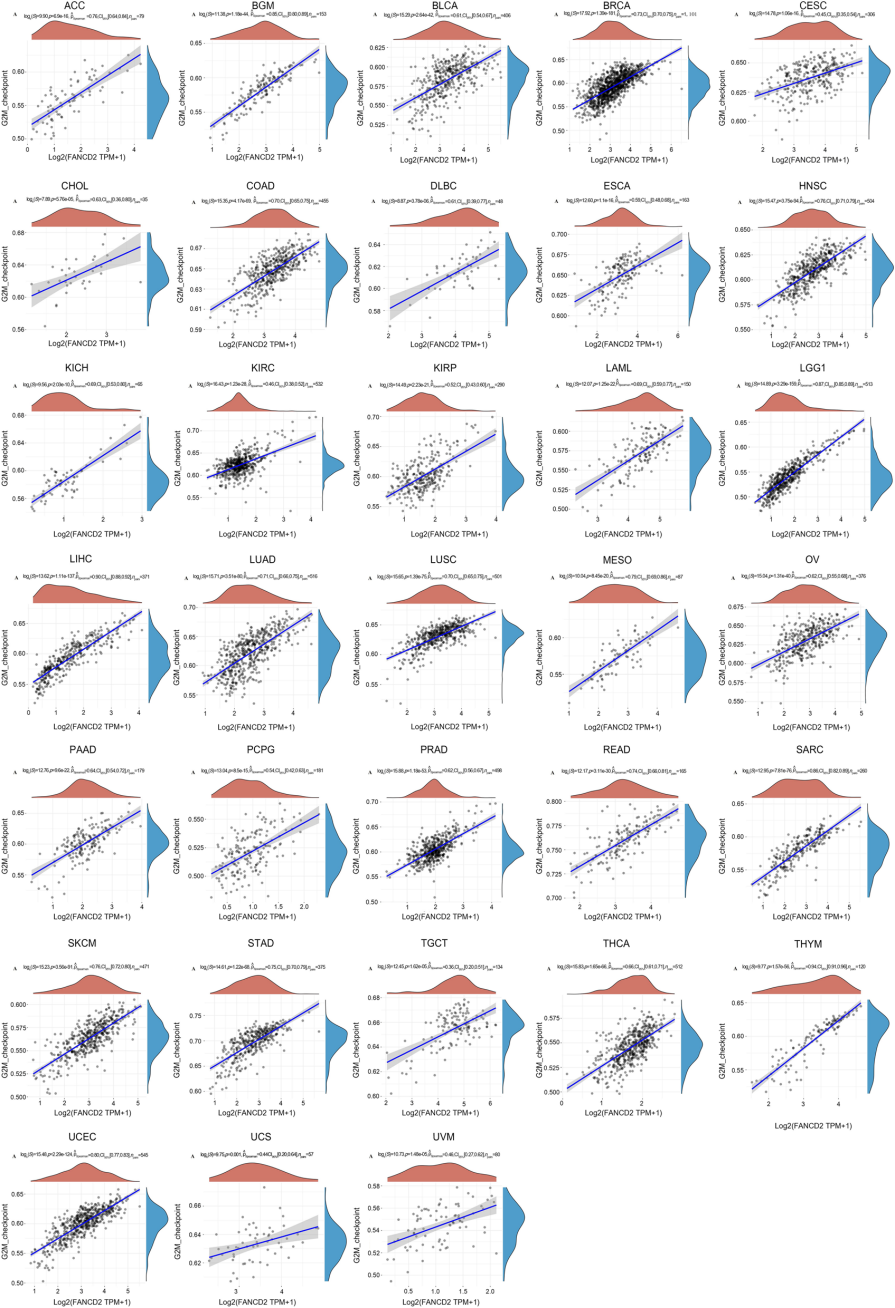
Correlation between *FANCD2* expression and G2/M checkpoint pathway in pan-cancer. *P < 0.05, **P < 0.01, and ***P < 0.001

**Fig. 9:**
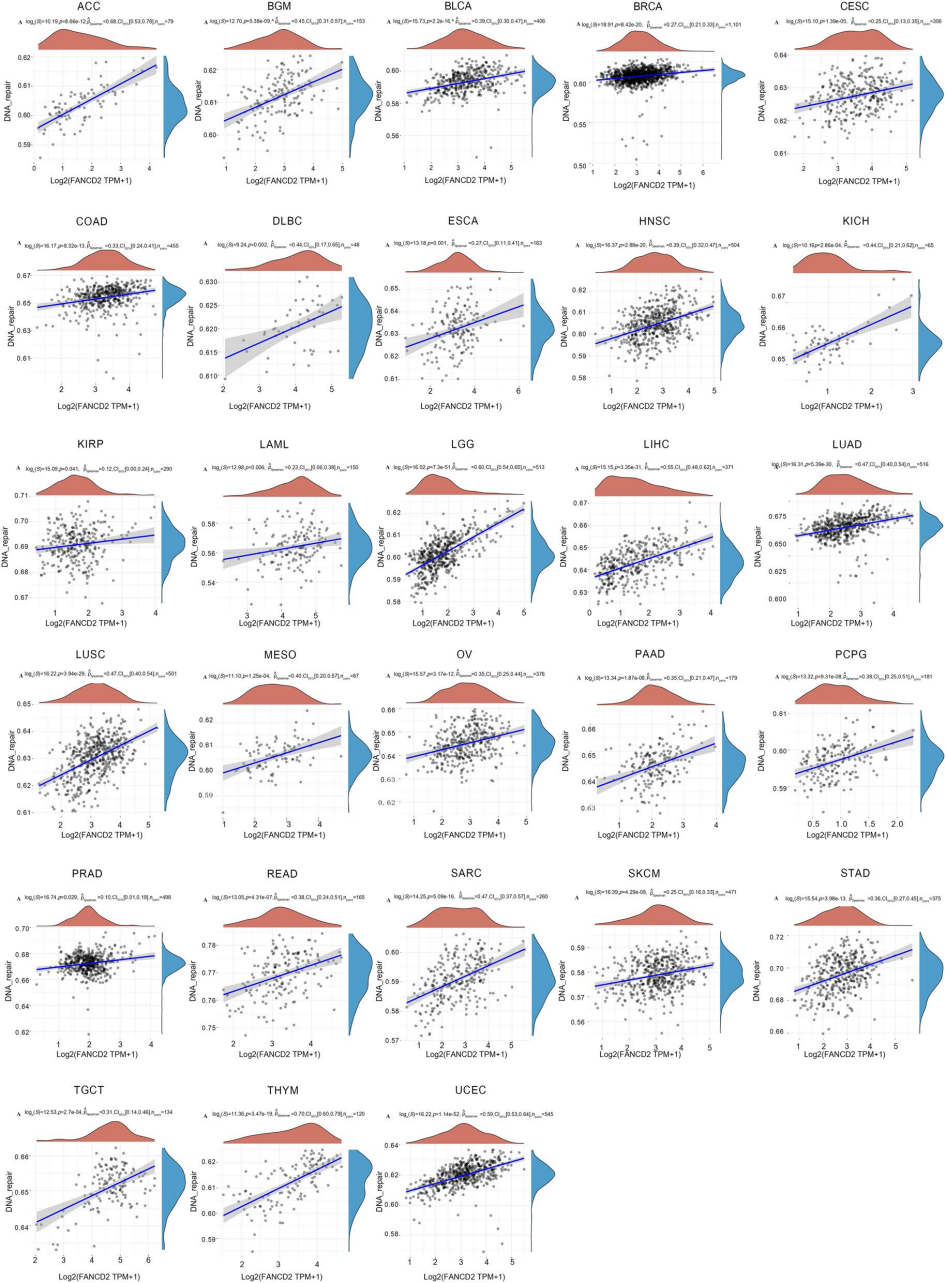
Correlation between *FANCD2* expression and DNA damage repair pathway in pan-cancer. *P < 0.05, **P < 0.01, and ***P < 0.001

The gene enrichment results demonstrated that cell cycle and DNA damage repair (DDR) pathways were closely associated with *FANCD2* tumorigenicity. Therefore, we conducted a more detailed examination of the relationship between *FANCD2* expression and the DDR pathway and G2/M checkpoint pathway. The findings demonstrated a significant association between *FANCD2* expression and these two pathways across various types of cancer. *FANCD2* expression was positively correlated with the G2/M pathway in 33 pan-carcinomas (Fig. 8). In addition, *FANCD2* expression was positively correlated with the DDR pathway in 28 cancers, except CHOL, KIRC, THCA, UCS, and UVM (Fig. 9).

## Discussion

As a crucial constituent of the FA pathway, the activation of *FANCD2* assumes the responsibility for rectifying interstrand crosslink (ICL) damage prompted by genotoxic stresses including ionizing radiation and DNA cross-linking agents, resolving DNA replication fork stalling, and upholding genomic stability [26]. While previous investigations have delineated the carcinogenic impact of *FANCD2* in diverse solid malignancies, limited knowledge exists regarding its expression and functionality in pan-cancer. Our preceding research has unveiled that impeding the monoubiquitination process and the formation of nuclear foci of FANCD2 could heighten the susceptibility of non-small cell lung cancer (NSCLC) to cisplatin-induced DNA damage and apoptosis [27]. Building upon the anticipated role of *FANCD2* in human malignancy, we have executed a comprehensive pan-cancer analysis aimed at characterizing the expression profile of *FANCD2* across diverse cancer types, while delving further into its prognostic potential, genetic alterations, association with the cancer immune microenvironment, and regulatory network. This endeavor seeks to foster an enhanced comprehension of the latent attributes of *FANCD2* in human cancers.

Based on our investigation utilizing the TCGA datasets, it was observed that the levels of *FANCD2* expression in tumor tissues were notably elevated in comparison to their corresponding normal tissues. This trend was observed in various types of cancers, including BLCA, BRCA, CESC, CHOL, COAD, ESCA, GBM, HNSC, KICH, KIRC, KIRP, LIHC, LUAD, LUSC, PCPG, PRAD, READ, SARC, STAD, THCA, and UCEC. Previous research has suggested that the amplification of *FANCD2* in esophageal squamous cell carcinoma leads to the emergence of a malignant nature, thereby promoting the progression of the tumor [28]. The heightened *FANCD2* expression has been correlated with an enlarged tumor size, a highly aggressive tumor phenotype, and an unfavorable prognosis in cases of hepatocellular carcinoma [15]. Additionally, *FANCD2* acts as an autonomous prognostic indicator for LUAD, exhibiting considerably elevated levels of expression in tumor tissues compared to their normal counterparts [29]. The results obtained from CPTAC and HPA database further substantiate our findings. Additionally, we observed a significant increase in FANCD2 expression with the progression of the disease in the majority of analyzed cancers. These discoveries indicate the potential of utilizing *FANCD2* as a diagnostic marker for specific types of tumors.

In our study, Cox regression analysis and Kaplan-Meier analysis demonstrated that high *FANCD2* expression was associated with a poor prognosis in a variety of tumors, which was consistent with the previous studies. Research has demonstrated that *FANCD2* possesses enhanced prognostic predicting efficacy in select malignancies. For instance, *FANCD2* can serve as a biomarker for prognosticating the likelihood of recurrence and survival in patients with nonmuscle invasive bladder cancer [30]. Elevated *FANCD2* expression acts as an independently unfavorable prognostic factor, exhibiting a positive correlation with tumor size and stage in spontaneous breast cancer [31]. These findings suggest that *FANCD2* may serve as a novel prognostic indicator in clinical applications.

The tumor microenvironment (TME) encompasses immune cells, tumor cells, and stromal microenvironment [32]. Among these components, tumor immune-infiltrating cells, constituting a significant portion of the TME, exhibit a close association with tumor progression, immune checkpoint inhibition, and immunotherapy [33–35]. In this study, we have examined the correlation between the expression of *FANCD2* and the infiltration of immune-related cells, revealing a close relationship between the level of immune cell infiltration and *FANCD2* expression across various cancer types. The maintenance of genomic stability is vital for cell survival and replication, as genomic instability may instigate cancer development, which escalates with the accumulation of DNA damage [36]. Notably, the DDR pathway, linked to tumor cells, greatly impacts immune surveillance, immune response, and immunogenicity [37]. The TME experiences perturbations due to augmented DNA damage and impaired repair [38]. Consequently, the DNA damage repair function of *FANCD2* may contribute to safeguarding genomic stability by influencing immune-infiltrating cells.

TMB has the potential to serve as a comprehensive biomarker across various cancers, aiding in the guidance of immunotherapy interventions [39]. Additionally, MSI can be utilized as a prognostic indicator for diverse malignancies, as well as a predictive factor for chemotherapy response and resistance in a wide array of tumor types [19, 40]. It has been observed that tumors exhibiting elevated TMB and MSI display enhanced sensitivity to immunotherapy [41, 42]. Our investigation has revealed a significant positive correlation between *FANCD2* expression and TMB in 12 tumors, as well as between *FANCD2* expression and MSI in 8 tumors. Furthermore, the expression of *FANCD2* showed significant correlations with various key immune checkpoint markers, such as CTLA4, HAVCR2, LAG3, PDCD1, PDCD1LG2, SIGLEC15, TIGIT, and CD274. These immune checkpoints operate as crucial mechanisms employed by tumors to evade immune responses [43]. Consequently, blockade of immune checkpoints hinders tumor immune evasion [44]. Immune checkpoint inhibitors (ICI) have emerged as remarkably effective in current cancer immunotherapy. By amalgamating these findings, we posit that *FANCD2* could serve as a novel and efficacious target for anti-cancer immunotherapy, complementing the use of chemotherapeutic agents.

GO and KEGG enrichment analysis revealed that *FANCD2*-related genes are involved in numerous potential pathways, particularly in cell cycle and DDR pathways. The DDR process plays a significant role in cancer susceptibility, progression, and response to treatment [45]. The FA pathway, which is activated in response to DNA damage, can maintain genomic stability during the DDR to prevent cancer [46]. Additionally, the FA pathway plays a critical role in cell division and helps protect chromosomes during mitosis [47]. In the G2/M phase of the cell cycle, FANCD2/FANCI protein dimers interconnect with sister chromatids and ensure proper chromosomal separation. This independent function of FANCD2/FANCI is a crucial checkpoint for tumor cells as they disproportionately depend on the G2/M checkpoint to avoid mitotic disasters [48, 49]. These groundbreaking discoveries align with previous research on post-translational modifications of FANCD2, such as phosphorylation and ubiquitination, which also participate intricately in DNA damage repair [50], regulation of the cell cycle [51], apoptosis [52], and chromatin remodeling [53]. Moreover, these modifications are closely associated with cellular growth, differentiation, and the maintenance of normal physiological functions within an organism.

Based on an analysis conducted using the cBioPortal database, it has been observed that alterations in the *FANCD2* gene are prevalent across various types of cancer. Among the 32 different cancer types examined, it was found that 11.92% of the BLCA population possessed mutations in the *FANCD2* gene, representing the highest occurrence rate among all cancer types. This finding suggests the importance of emphasizing the clinical significance of *FANCD2* gene mutations and the potential for targeted therapy in the context of BLCA. Furthermore, a comprehensive assessment was carried out to investigate the relationship between *FANCD2* expression levels and disease-specific survival or progression-free intervals in cancer patients. The analysis demonstrated that, in general, *FANCD2* could exhibit adverse effects on overall survival, disease-specific survival, or progression-free intervals in some cancer cases. DNA methylation, a prevailing form of epigenetic alteration, governs cellular essence by regulating gene manifestation and genetic equilibrium, whilst deviant DNA methylation has the potential to foster carcinogenesis [54]. Through scrutinizing the epigenetic blueprint of *FANCD2* in 33 malignancies, we have unearthed that abnormal levels of DNA methylation could potentially precipitate atypical expression of *FANCD2* within tumors. This peculiar phenomenon may be attributed to two primary factors. Firstly, the overmethylation of promoter regions may instigate the suppression of tumor suppressor genes, thereby modulating diverse regulatory proteins and enzymes. Secondly, in the nascent stages of cancer progression, hypomethylation might facilitate genomic instability and cellular metamorphosis [55].

While we have examined data from various databases, it is important to acknowledge certain limitations in this study. Firstly, although bioinformatic analyses have offered valuable insights into the role of *FANCD2* in pan-cancer, it is necessary to conduct further experiments to validate these findings. Secondly, even though our study has demonstrated a correlation between *FANCD2* expression and immune activity as well as clinical survival in pan-cancer, we cannot definitively confirm whether *FANCD2* directly impacts clinical survival through an immune pathway.

In conclusion, our comprehensive analysis of pan-cancer data has shed light on the potential involvement of *FANCD2* in a wide range of cancer types. Based on our findings, we propose that *FANCD2* holds promise as a novel diagnostic biomarker and therapeutic target for cancers such as lung, breast, liver, and colon cancer, among others. Moreover, our research advances the understanding of the role of *FANCD2* in cancer immunotherapy, as we have observed significant associations between *FANCD2*, immune cells, and immune checkpoints. Moving forward, conducting further experiments and prospective studies on *FANCD2* in diverse cancer types can provide valuable insights into its regulatory mechanisms and contribute to the development of targeted therapeutic strategies.

### Supplementary Information

**Supplementary Material 1.**

## Data Availability

The data used to support the findings of this study are available from the corresponding author upon request.

## References

[1] Siegel RL, Miller KD, Wagle NS (2023). Cancer statistics, 2023. CA Cancer J Clin.

[2] Bray F, Ferlay J, Soerjomataram I (2018). Global cancer statistics 2018: GLOBOCAN estimates of incidence and mortality worldwide for 36 cancers in 185 countries. CA Cancer J Clin.

[3] Chen S, Cao Z, Prettner K, Kuhn M (2023). Estimates and projections of the Global Economic cost of 29 cancers in 204 countries and territories from 2020 to 2050. JAMA Oncol.

[4] Carrera PM, Kantarjian HM, Blinder VS (2018). The financial burden and distress of patients with cancer: understanding and stepping-up action on the financial toxicity of cancer treatment. CA Cancer J Clin.

[5] Lin L, Li Z, Yan L (2021). Global, regional, and national cancer incidence and death for 29 cancer groups in 2019 and trends analysis of the global cancer burden, 1990–2019. J Hematol Oncol.

[6] Mun EJ, Babiker HM, Weinberg U (2018). Tumor-treating fields: a fourth modality in Cancer Treatment. Clin Cancer Res.

[7] Tsimberidou AM, Fountzilas E, Nikanjam M (2020). Review of precision cancer medicine: evolution of the treatment paradigm. Cancer Treat Rev.

[8] Sung H, Ferlay J, Bray F (2021). Global cancer statistics 2020: GLOBOCAN estimates of incidence and mortality worldwide for 36 cancers in 185 countries. CA Cancer J Clin.

[9] Ceccaldi R, Sarangi P, D’Andrea AD (2016). The fanconi anaemia pathway: new players and new functions. Nat Rev Mol Cell Biol.

[10] Tsui V, Crismani W (2019). The Fanconi Anemia Pathway and Fertility. Trends Genet.

[11] Nepal M, Che R, Zhang J (2017). Fanconi Anemia Signaling and Cancer. Trends Cancer.

[12] Badra Fajardo N, Taraviras S, Lygerou Z (2022). Fanconi anemia proteins and genome fragility: unraveling replication defects for cancer therapy. Trends Cancer.

[13] Okamoto Y, Iwasaki WM, Kugou K (2018). Replication stress induces accumulation of *FANCD2* at central region of large fragile genes. Nucleic Acids Res.

[14] Zheng C, Ren Z, Chen H (2022). *FANCD2* promotes the malignant behavior of endometrial cancer cells and its prognostic value. Exp Cell Res.

[15] Komatsu H, Masuda T, Iguchi T (2017). Clinical significance of *FANCD2* gene expression and its Association with Tumor Progression in Hepatocellular carcinoma. Anticancer Res.

[16] Moes-Sosnowska J, Rzepecka IK, Chodzynska J (2019). Clinical importance of *FANCD2*, *BRIP1*, *BRCA1, BRCA2* and *FANCF* expression in ovarian carcinomas. Cancer Biol Ther..

[17] Zhang X, Li L, Huang S (2022). Comprehensive Analysis of ANLN in Human tumors: a prognostic Biomarker Associated with Cancer Immunity. Oxid Med Cell Longev.

[18] Thorsson V, Gibbs DL, Brown SD (2018). The Immune Landscape of Cancer. Immunity.

[19] Bonneville R, Krook MA, Kautto EA (2017). Landscape of Microsatellite Instability Across 39 Cancer Types. JCO Precis Oncol.

[20] Zhang Y, Zhang Z (2020). The history and advances in cancer immunotherapy: understanding the characteristics of tumor-infiltrating immune cells and their therapeutic implications. Cell Mol Immunol.

[21] Safarzadeh A, Alizadeh M, Beyranvand F (2021). Varied functions of immune checkpoints during cancer metastasis. Cancer Immunol Immunother.

[22] DeStefanis RA, Kratz JD, Emmerich PB (2019). Targeted therapy in metastatic colorectal cancer: current standards and novel agents in review. Curr Colorectal Cancer Rep.

[23] Wu Y, Zhang J, Hou C (2022). A Pancancer Study of *PIEZO1* as a prognosis and Immune Biomarker of Human tumors. J Oncol.

[24] Meng H, Cao Y, Qin J (2015). DNA methylation, its mediators and genome integrity. Int J Biol Sci.

[25] Papanicolau-Sengos A, Aldape K (2022). DNA methylation profiling: an emerging paradigm for Cancer diagnosis. Annu Rev Pathol.

[26] Jacquemont C, Taniguchi T (2007). The fanconi anemia pathway and ubiquitin. BMC Biochem.

[27] Fan XZ, Chen YF, Zhang SB (2021). Centipeda minima extract sensitizes lung cancer cells to DNA-crosslinking agents via targeting fanconi anemia pathway. Phytomedicine.

[28] Lei LC, Yu VZ, Ko JMY (2020). *FANCD2* confers a malignant phenotype in esophageal squamous cell carcinoma by regulating cell cycle progression. Cancers (Basel).

[29] Miao H, Ren Q, Li H (2022). Comprehensive analysis of the autophagy-dependent ferroptosis-related gene *FANCD2* in lung adenocarcinoma. BMC Cancer.

[30] Roh YG, Mun JY, Kim SK (2020). Fanconi Anemia Pathway activation by *FOXM1* is critical to bladder Cancer recurrence and Anticancer Drug Resistance. Cancers (Basel).

[31] Feng L, Jin F (2019). Expression and prognostic significance of fanconi anemia group D2 protein and breast cancer type 1 susceptibility protein in familial and sporadic breast cancer. Oncol Lett.

[32] Pansy K, Uhl B, Krstic J (2021). Immune Regulatory processes of the Tumor Microenvironment under Malignant conditions. Int J Mol Sci.

[33] Hinshaw DC, Shevde LA (2019). The Tumor Microenvironment innately modulates Cancer Progression. Cancer Res.

[34] Lv B, Wang Y, Ma D (2022). Immunotherapy: reshape the Tumor Immune Microenvironment. Front Immunol.

[35] Petitprez F, Meylan M, de Reyniès A (2020). The Tumor Microenvironment in the response to Immune Checkpoint Blockade therapies. Front Immunol.

[36] Huang R, Zhou PK (2021). DNA damage repair: historical perspectives, mechanistic pathways and clinical translation for targeted cancer therapy. Signal Transduct Target Ther.

[37] Shi C, Qin K, Lin A (2022). The role of DNA damage repair (DDR) system in response to immune checkpoint inhibitor (ICI) therapy. J Exp Clin Cancer Res.

[38] Sun W, Zhang Q, Wang R (2021). Targeting DNA damage repair for Immune Checkpoint Inhibition: mechanisms and potential clinical applications. Front Oncol.

[39] Fumet JD, Truntzer C, Yarchoan M, Ghiringhelli F (2020). Tumour mutational burden as a biomarker for immunotherapy: current data and emerging concepts. Eur J Cancer.

[40] Baretti M, Le DT (2018). DNA mismatch repair in cancer. Pharmacol Ther.

[41] Filipovic A, Miller G, Bolen J (2020). Progress toward identifying exact proxies for Predicting Response to Immunotherapies. Front Cell Dev Biol.

[42] Chang L, Chang M, Chang HM (2018). Microsatellite instability: a predictive biomarker for Cancer Immunotherapy. Appl Immunohistochem Mol Morphol.

[43] Davern M, Donlon NE, O’ Connell F (2022). Cooperation between chemotherapy and immune checkpoint blockade to enhance anti-tumour T cell immunity in oesophageal adenocarcinoma. Transl Oncol.

[44] Ma C, Sun X, Shen D (2020). Ectopic expression of *LAG-3* in non-small-cell lung cancer cells and its clinical significance. J Clin Lab Anal.

[45] Ding K, He Y, Wei J (2022). A score of DNA damage repair pathway with the predictive ability for chemotherapy and immunotherapy is strongly associated with immune signaling pathway in pan-cancer. Front Immunol.

[46] Che R, Zhang J, Nepal M (2018). Multifaceted Fanconi Anemia Signaling. Trends Genet.

[47] Nalepa G, Clapp DW (2018). Fanconi anaemia and cancer: an intricate relationship. Nat Rev Cancer.

[48] Schmidt M, Rohe A, Platzer C (2017). Regulation of G2/M transition by inhibition of WEE1 and PKMYT1 kinases. Molecules.

[49] Chan KL, Palmai-Pallag T, Ying S (2009). Replication stress induces sister-chromatid bridging at fragile site loci in mitosis. Nat Cell Biol.

[50] Zhao S, Huang C, Yang Y (2023). DNA repair protein FANCD2 has both ubiquitination-dependent and ubiquitination-independent functions during germ cell development. J Biol Chem.

[51] Cantres-Velez JA, Blaize JL, Vierra DA (2021). Cyclin-dependent kinase-mediated phosphorylation of FANCD2 promotes Mitotic Fidelity. Mol Cell Biol.

[52] Bao Y, Feng H, Zhao F (2021). *FANCD2* knockdown with shRNA interference enhances the ionizing radiation sensitivity of nasopharyngeal carcinoma CNE-2 cells. Neoplasma.

[53] Hejna J, Holtorf M, Hines J (2008). Tip60 is required for DNA interstrand cross-link repair in the fanconi anemia pathway. J Biol Chem.

[54] Nishiyama A, Nakanishi M (2021). Navigating the DNA methylation landscape of cancer. Trends Genet.

[55] Martisova A, Holcakova J, Izadi N (2021). DNA methylation in solid tumors: functions and methods of detection. Int J Mol Sci.

