# Correction: Pancancer analysis of the prognostic and immunological role of FANCD2: a potential target for carcinogenesis and survival

**DOI:** 10.1186/s12920-024-01896-6

**Published:** 2024-05-14

**Authors:** Zedan Zhao, Ruyu Wang, Ruixue Wang, Jialing Song, Fengjun Ma, Huafeng Pan, Cuiyun Gao, Deqiang Wang, Xuemei Chen, Xiangzhen Fan

**Affiliations:** 1 Department of Rehabilitation Medicine, Binzhou Medical University Hospital, Binzhou, Shandong 256603 China; 2 School of Rehabilitation Medicine, Binzhou Medical University, Yantai, Shandong 264003 China; 3 Department of Obstetrics, Binzhou Medical University Hospital, Binzhou, Shandong 256603 China; 4 School of clinical medicine, Jiangxi University of Chinese Medicine, Nanchang, Jiangxi 330004 China; 5 Shandong University of Traditional Chinese Medicine, Jinan, Shandong China; 6 Science and Technology Innovation Center, Guangzhou University of Chinese Medicine, Guangzhou, Guangdong China

**Correction to: Zhao et al. BMC Medical Genomics (2024) 17:69**

10.1186/s12920-024-01836-4

Following the publication of the original article, the authors reported an incorrect funding statement. The correct grant number have been “No. ZR2022QH001”, not “No. ZR202111290609” as shown in the published version.

Thus, the correct funding note is as follows: This work was supported by the China Postdoctoral Science Foundation (grant no. 2023M732138), the Natural Science Foundation of Shandong Province (grant no. ZR2022QH001) and the Science and Technology Project of Binzhou Medical University (grant no. BY2021KYQD33). All fundings contribute to the design of the study and collection, analysis, and interpretation of data and in writing the manuscript.

The original article has been corrected.

