# Supplementary material for: Pancancer analysis of the prognostic and immunological role of FANCD2: a potential target for carcinogenesis and survival
Source: BMC Med Genomics. 2024 Mar 5;17:69. doi: 10.1186/s12920-024-01836-4 (PMC10916239; doi:10.1186/s12920-024-01836-4)
Supplement: Supplementary file 1 — Supplementary Material 1. [file 12920_2024_1836_MOESM1_ESM.docx]

**Supplementary Materials**


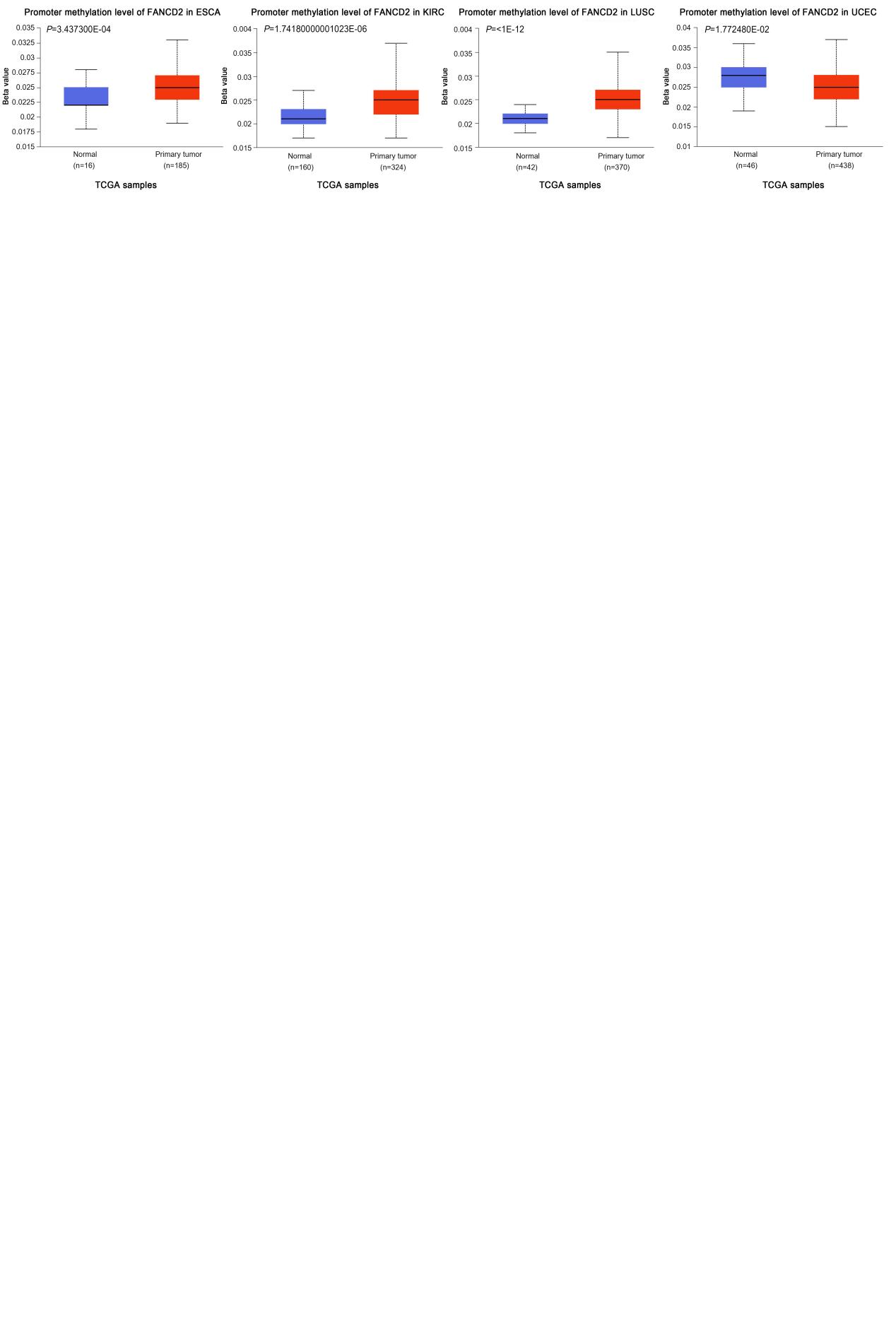


Supplementary Figure 1: Promoter methylation levels of *FANCD2* in pan-cancer obtained from the UALCAN database. **P* <0.05, ***P* < 0.01, and ****P* < 0.001.
